# Pinin associates with prognosis of hepatocellular carcinoma through promoting cell proliferation and suppressing glucose deprivation-induced apoptosis

**DOI:** 10.18632/oncotarget.9233

**Published:** 2016-05-09

**Authors:** Xuejun Yang, Deguang Sun, Chengyong Dong, Yu Tian, Zhenming Gao, Liming Wang

**Affiliations:** ^1^ Department of General Surgery, The First Affiliated Hospital of Dalian Medical University, Dalian 116027, China; ^2^ Department of General Surgery, The Second Affiliated Hospital of Dalian Medical University, Dalian 116011, China

**Keywords:** hepatocellular carcinoma, Pinin, proliferation, apoptosis, ERK

## Abstract

The roles of Pinin have been well studied in epithelial cell-cell adhesion and RNA alternative splicing, which suggests its involvement in cancer progression. However, little is known about the association between Pinin expression and hepatocellular carcinoma (HCC) tumorigenesis. In this study we report increased expression of Pinin in HCC tissues and cells. Elevated levels of Pinin closely associates with pathological grade and overall survival of patients with hepatocellular carcinoma. Suppression of Pinin expression via lentivirus mediated shRNA knockdown inhibits HCC cell proliferation, colony formation, cell viability, but promotes glucose deprivation (GD)-induced cell apoptosis. On the contrary, overexpression of Pinin reverses these effects observed in Pinin depleted cells. Meanwhile, overexpression of Pinin attenuates GD initiated poly ADP-ribose polymerase (PARP) cleavage and ERK1/2 dephosphorylation, which can be completely blocked with MEK1/2 inhibitor U0126. Therefore, we conclude that Pinin contributes to HCC progression and resistance to GD-induced apoptosis via maintaining ERK1/2 activation and hence may be a potential therapeutic target in hepatocellular carcinoma treatment.

## INTRODUCTION

Liver cancer is the fifth most commonly diagnosed cancer and the second leading cause of cancer deaths in the world [1, 2]. Among different liver cancer subtypes, hepatocellular carcinoma (HCC) is the most malignant and major histological type, which accounts for 70–85% of primary liver cancer in the clinic [3, 4]. Given the fact that HCC has extremely low 5-year survival rate and causes approximately 750,000 deaths each year [5, 6], novel markers for diagnosis with benefits for HCC risk assessment as well as targets for better management of HCC are urgently required.

The activation of oncogenes or loss of tumor suppressors often leads to altered cellular metabolism, which is considered as a hallmark of cancer and plays a key role in hepatocarcinogenesis [7, 8]. It is well known that the Warburg effect with characteristics of enhanced glucose uptake and lactate production, is one of the most important metabolic transformation during tumorigenesis [9, 10]. Additionally, up regulation of glutamine and fatty acid metabolism is also frequently observed in tumor cells [11, 12]. Therefore, revelation of these metabolic adaptations may contribute to the exploitation of potential therapeutic strategies.

Pinin, a serine/arginine-rich (SR)-related protein, is a dual location protein found either in desmosomes facilitating cell-cell adhesion or in the nucleus regulating pre-mRNA alternative splicing and export. It is originally identified in the desmosome plaque acting as an associated protein for intermediate filaments or the maintenance of epithelial cell-cell adhesions in small intestine and cornea [13–19]. In various human cancers, the expression and bio-functions of Pinin have been gradually disclosed. Firstly, Pinin was reported to be a potential tumor suppressor in renal cell carcinoma and increased Pinin expression inhibited cell anchorage independent growth [20]. Subsequently, Pinin was observed to promote cell growth and survival via upregulating BCL-xL expression in human breast cancer cells. However, the role of Pinin in HCC is largely unknown.

In the present study, we report that Pinin expression is significantly increased in HCC tissues and cells. Elevation of Pinin closely associates with pathological grades and overall survival of patients with hepatocellular carcinoma. Furthermore, the suppression of Pinin reduced HCC cells proliferation, colony formation, and cell viability. Meanwhile, downregulation of Pinin expression inhibited ERK1/2 phosphorylation and promoted glucose deprivation (GD)-induced cell apoptosis.

## RESULTS

### Pinin is overexpressed in human HCC cell lines and tissues

**Figure 1:**
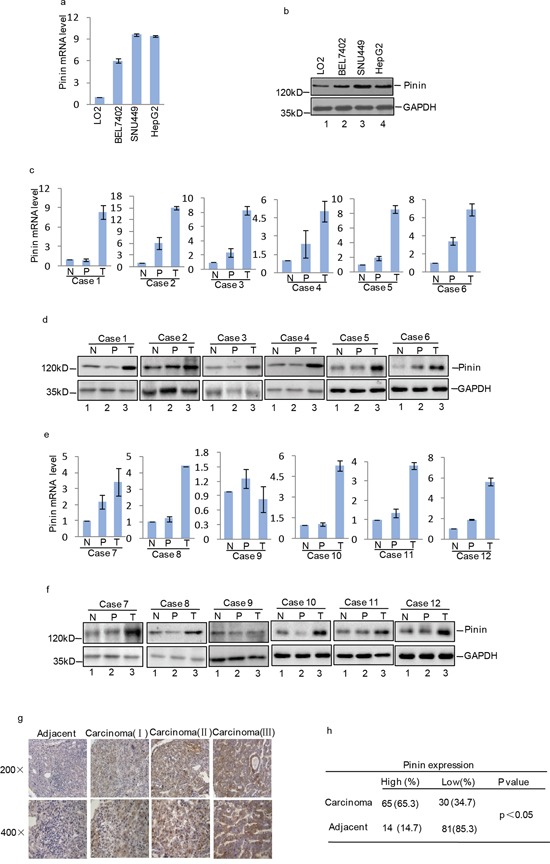
Expression of Pinin in normal human liver tissues, HCC tissues, normal human liver cell line and HCC cell lines **a.** Pinin mRNA expression level in normal human liver cell LO2 and HCC cell lines (BEL7402, SNU449 and HepG2) was detected by q-RT-PCR (n=3, mean ± SD, *t*-test, **P<0.01, ***P<0.001 vs. LO2). **b.** Pinin protein expression level in normal human liver cell LO2 and HCC cell lines (BEL7402, SNU449 and HepG2) was detected by western blot. **c-f.** Pinin mRNA and protein expression level in normal liver tissues (N), peritumoral tissues (P) and tumors (T) from HCC patients (n=3, mean ± SD, *t*-test, **P<0.01, ***P<0.001 vs. N. *P<0.05,**P<0.01, ***P<0.001 vs. P). **g-h.** Pinin protein level in peritumoral and carcinoma tissues was detected by immunohistochemical staining.

To investigate differential expression of Pinin between HCC cells and non-carcinoma cells, human normal liver cell line LO2 and other three carcinoma cell lines (BEL7402, SNU449 and HepG2) were analyzed. We found that Pinin mRNA (Figure 1a) and protein (Figure 1b) expression level was remarkably increased in HCC cell lines. Notably, similar increase was detected in HCC tissues in comparison with adjacent normal tissues (Figure 1c-1f). Inimmunohistochemical analyses with 95 human HCC samples, 62 cases (65.3%) show overexpression of Pinin in HCC tissues as compared to the corresponding peritumoral tissues. In these clinical samples, moderate to strong cytoplasmic staining was observed at different clinical stages (Figure 1g, 1h).

**Figure 2:**
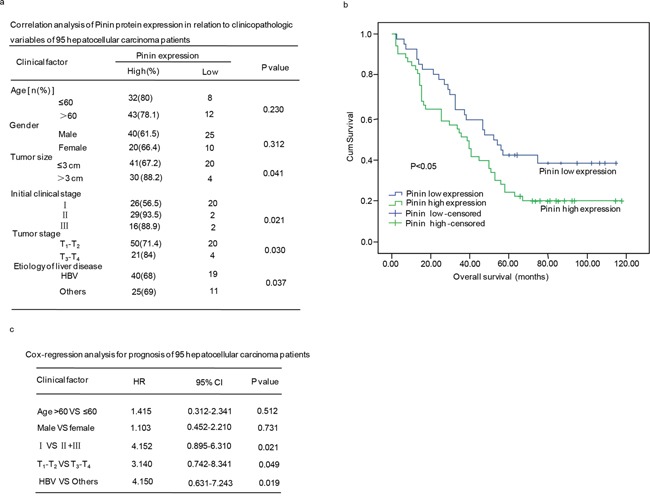
The clinical correlation analyses of Pinin protein expression in 95 hepatocellular carcinoma patients **a.** Correlation analyses of Pinin protein expression in relation to clinicopathologic variables of 95 hepatocellular carcinoma patients. **b.** Kaplan-Meier analysis of overall survival with high or low Pinin expression. **c.** Cox-regression analyses fro prognosis of 95 patients with hepatocellular carcinoma.

We further investigated the correlation between Pinin expression and clinicopathological parameters, including age, gender, tumor size, clinical stage, and tumor stage. We observed statistically significant correlations between Pinin expression and tumor size (p=0.041), clinical stage (p=0.021), tumor stage (p=0.030) and etiology of liver disease (p=0.037). No significant association was found between Pinin expression and the other clinicopathological variables including age (p=0.230) and gender (p=0.312) (Figure 2a).

Additionally, a significant correlation between Pinin expression and overall survival of 95 hepatocellular carcinoma patients was observed, which revealed that patients with low Pinin expression showed significant higher overall survival rate as compared to those patients with high Pinin expression (p<0.05) (Figure 2b). The Cox multivariate regression analysis with clinicopathological factors was performed to assess independent prognostic factor of overall survival. Pinin expression was found to be an independent prognostic factor for the survival of hepatocellular carcinoma patients (Figure 2c).

### Pinin promotes clonogenicity and proliferation of HCC cells

**Figure 3:**
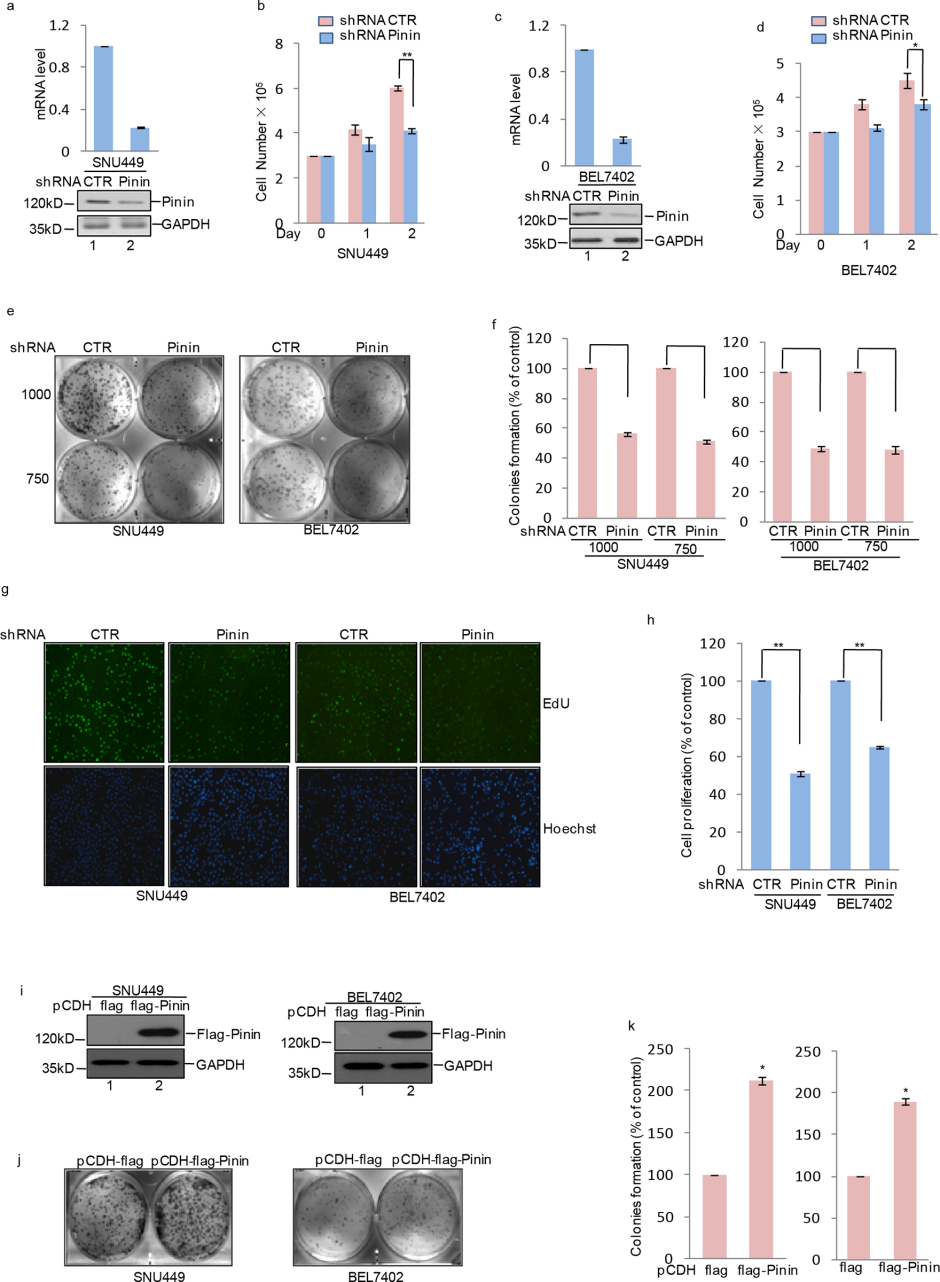
The effects of Pinin on clonogenicity and proliferation in HCC cells **a, c.** shRNA-mediated silencing of Pinin expression in human HCC cell lines. Western blot and q-RT-PCR detected the expression of Pinin in SNU449 and BEL7402 cells. **b, d.** Cells with or without knockdown of Pinin were cultured for the days as indicated and cell growth was evaluated by trypan blue staining (n=3, mean ± SD, *t*-test, *P<0.05, **P<0.01 vs. shRNA CTR). **e, f.** Colony formation assay was used to measure the clonogenicity of SNU449 and BEL7402 cells with or without knockdown of Pinin. Cells were grown in the same condition. All dishes were fixed, stained and photographed at the same time. The number of untreated cells were set as 100% (n=3, mean ± SD, *t*-test, **P<0.01 vs. shRNA CTR). **g, h.** SNU449 and BEL7402 cells with or without knockdown of Pinin were stained with EdU. The nuclei were also stained by Hoechst 33342. The percentage of cell proliferation was expressed as the ratio of EdU positive cells to total Hoechst 33342 positive cells. The number of untreated cells were set as 100% (n=3, mean ± SD, *t*-test, **P<0.01 vs. shRNA CTR). **i-k.** Colony formation assay was used to measure the clonogenicity of SNU449 and BEL7402 cells with or without overexpression of Pinin. Cells were grown in the same condition. All dishes were fixed, stained and photographed at the same time. Western blot detected the protein level of Pinin using the antibodies as indicated. The number of untreated cells was set as 100%. (n=3, mean ± SD, *t*-test, *P<0.05 vs. pCDH-flag).

In order to evaluate the importance of Pinin in regulating biological processes in HCC cells, we firstly suppressed its endogenous expression in SNU449 and BEL7402 cells with shRNA medicated knockdown (Figure 3a, 3c). Depletion of Pinin led to inhibition of cell proliferation (Figure 3b, 3d) and colony formation (Figure 3e, 3f), as well as decrease in DNA synthesis (Figure 3g, 3h) in HCC cell lines. On the contrary, an increase in colony formation was observed in SNU449 and BEL7402 cells (Figure 3j, 3k) with exogenous overexpression of Flag labeled Pinin (Figure 3i). Taken together, Pinin exhibits a role in promoting clonogenicity and proliferation of HCC cells and thus demonstrates a positive impact on this key pathway for tumorogensis.

### Pinin inhibits glucose deprivation-induced apoptosis in HCC cells

**Figure 4:**
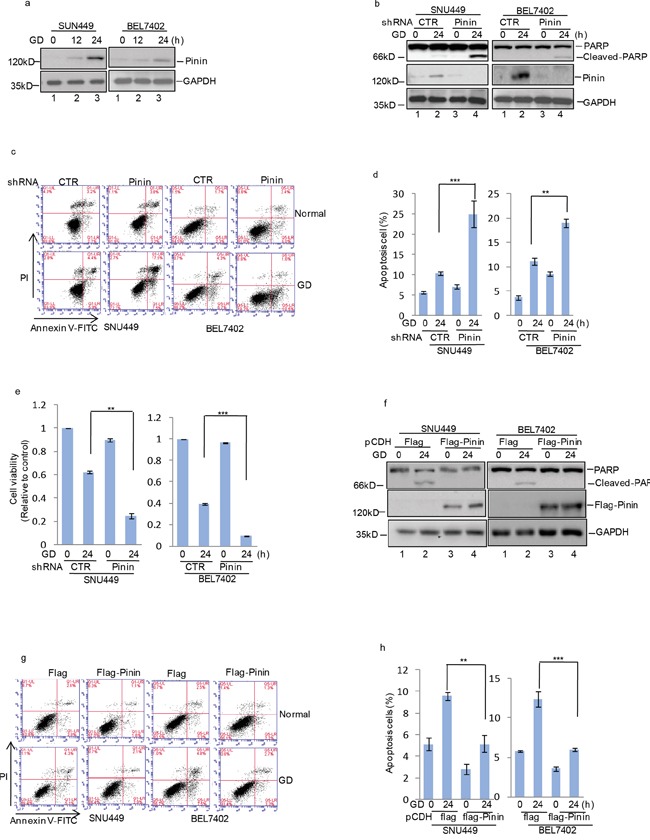
Pinin inhibits glucose deprivation–induced apoptosis in HCC cells **a.** SNU449 and BEL7402 cells were cultured with or without glucose deprivation for the indicated periods. Cell lysates were separated by SDS-PAGE and analyzed by western blot with indicated antibodies. GAPDH was used as the loading control. **b.** SNU449 and BEL7402 cells with or without knockdown of Pinin were cultured in present of glucose or not for 24 h. GAPDH was used as the loading control. **c, d.** The percentage of cell apoptosis was analyzed by flow cytometry analysis. (n=3, mean ± SD, *t*-test, **P<0.01, ***P<0.001 vs. shRNA CTR). **e.** Cell viability was determined by CCK8 assay.(n=3, mean ± SD, *t*-test, **P<0.01, ***P<0.001 vs. shRNA CTR). **f.** SNU449 and BEL7402 cells with or without overexpression of Pinin were cultured in present of glucose or not for 24 h. Cell lysates were then subjected to western blot analysis with the antibodies indicated. GAPDH was detected as the loading control. **g, h.** The percentage of cell apoptosis was analyzed by flow cytometry analysis. (n=3, mean ± SD, *t*-test, **P<0.01, ***P<0.001 vs. pCDH-flag).

Energy consumption and metabolism are central events for governing cell behavior, especially glucose utilization, which controls tumor cell growth and survival. Due to the high rate of glycolysis and inadequate vascularization, solid tumors usually grow under conditions constantly depleted of oxygen and crucial nutrients, particularly glucose [3, 7, 21]. To investigate the role of Pinin in HCC cell survival during metabolic stress, SNU449 cells transfected with Pinin expressing or control vectors were cultured in medium deprived of glucose, glutamine, or fetal bovine serum (FBS) to mimic different metabolic stress conditions. Notably, compared with control cells, Pinin overexpressing cells were more resistant to cell death induced by glucose depletion, whereas no significant protective effect of Pinin overexpression was observed in SNU449 cells under conditions of glutamine or serum deprivation (Supplementary Figure 1a-1c). Additionally, we also investigated the influence of GD treatment on endogenous Pinin expression. A gradual increase in Pinin expression in both SNU449 and BEL7402 cells was observed during 24 h of glucose starvation (Figure 4a). Furthermore, knockdown of Pinin expression in HCC cells (Figure 4b) increased the proportion of apoptotic cells (Figure 4c, 4d) and reduced cell viability (Figure 4e) following glucose deprivation, which was accompanied with enhanced PARP cleavage (Figure 4b). Conversely, exogenously expressed Flag-tagged Pinin efficiently suppressed these GD effects (Figure 4g, 4h) on cell apoptosis as well as PARP activation (Figure 4f).

### Pinin maintains ERK activation in HCC cells under glucose deprivation

**Figure 5:**
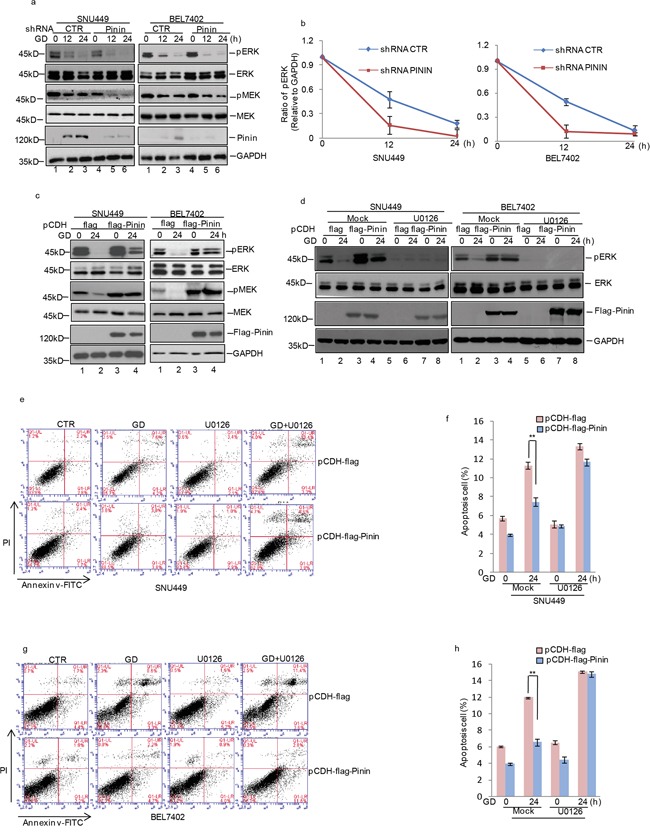
Effects of ERK inactivation on Pinin modulated HCC apoptosis during glucose deprivation **a.** SNU449 and BEL7402 cells with or without knockdown of Pinin were cultured in present of glucose or not for the indicated periods. Cell lysates were analyzed with the indicated antibodies. GAPDH was detected as the loading control. **b.** Quantification of pERK levels relative to GAPDH is shown. **c.** Pinin was overexpressed in SNU449 and BEL7402 cells and then the cells were cultured in present of glucose or not for 24 h. Cell lysates were analyzed with the antibodies indicated. GAPDH was detected as the loading control. **d.** Pinin was overexpressed in SNU449 and BEL7402 cells and then the cells were cultured in present of glucose or not for 24h with or without U0126 treatment. Cell lysates were analyzed with the antibodies indicated. GAPDH was detected as the loading control. **e-h.** The percentage of cell apoptosis was analyzed by flow cytometry analysis in SNU449 and BEL7402 cells. (n=3, mean ± SD, *t*-test, **P<0.01 vs. pCDH-flag).

Due to the pivotal role of MAPK/ERK pathway in regulating growth and apoptosis of HCC cells, we examined whether Pinin participated in controlling ERK1/2 activation. It was observed that ERK1/2 and MEK phosphorylation was gradually reduced under GD condition at indicated time and became nearly undetectable at 24 h (Figure 5a, 5c). Moreover, in Pinin depleted cells at 12 h of GD treatment, a significant decrease in ERK and MEK activation was observed as compared with control cells (Figure 5a, 5b). However, opposite effects on ERK and MEK phosphorylation were observed in cells with exogenous Pinin overexpression (Figure 5c and Supplementary Figure 2a, 2b). Furthermore, U0126, a specific inhibitor of MEK1/2, was used to assess the effects of Pinin on ERK1/2 activation. We observed that Pinin mediated resistance to GD-induced ERK dephosphorylation (Figure 5d) was totally blocked by U0126. In addition, exogenous Pinin overexpression did not significantly reduce GD-induced apoptosis in cells treated with U0126 (Figure 5e-5h).

## DISCUSSION

Pinin is a dual location protein found either in the nucleus regulating pre-mRNA alternative splicing and export or in desmosomes mediating cell-cell adhesions [16, 17, 19]. The biological functions of Pinin have been mainly discovered in epithelial cell behaviors as its participation in forming desmosome. Besides, its aberrant expression suppressed renal cancer development [14, 17].

In this study, we report increased Pinin mRNA and protein expression levels in HCC tissues and cell lines. Elevation of Pinin levels closely associated with pathological grade and overall survival of patients with hepatocellular carcinoma. Subsequently, loss and gain of expression approaches were applied to modulate Pinin expression levels in SNU449 and BEL7402 cells. By series of functional assays, we identified Pinin as an oncogenic proteinin HCC cells as efficient knockdown of Pinin expression resulted in a significant reduction in proliferation, colony formation and DNA synthesis of HCC cells. On the contrary, when we overexpressed exogenous Pinin, the ability of HCC cells to form colonies was greatly promoted. Of note, Shi and colleagues demonstrated that Pinin acted as a tumor suppressor due to low expression in renal carcinoma. Overexpression of Pinin impaired J82 and EcR-293 cell growth in soft agar [22]. These controversial observations might be due to that Pinin regulates different downstream factors and the various genetic backgrounds between renal carcinoma and HCC.

Owing to the high rate of glycolysis and inadequate vascularization, HCC usually grows under conditions constantly depleted of oxygen and crucial nutrients, particularly glucose [23]. Considering the critical role of glucose in tumor metabolism, we further investigated the involvement of Pinin in survival and apoptosis of HCC cells under glucose deprived conditions. We performed experiments to evaluate the influence of Pinin on GD-induced cell apoptosis. Our data indicated that GD treatment effectively induced apoptosis in HCC cells, which may be explained by reduction in cell viability, with similar phenomena previously observed in breast cancer, colon cancer and ovarian cancer [24–26]. Furthermore, HCC cells with decreased Pinin expression exhibited a moderate increase in apoptosis rate in culture condition with normal glucose (25 mM). Consistently, significantly elevated apoptosis was triggered by GD in Pinin depleted HCC cells, suggesting an important role of Pinin in regulating GD induced apoptosis of HCC cells. Conversely, exogenous overexpression of Pinin was able to competently antagonize GD-induced apoptosis. These results demonstrated the important role of Pinin in the regulation of cell apoptosis in HCC, especially under GD condition. Recently, Steve Leu reported that Pinin regulated alternative splicing of BCL-xL [19], a key pro or anti-apoptotic factor in MCF7 cells and loss of Pinin might lead to cell apoptosis. However, we were unable to detect the altered BCL-xL expression in HCC after modulating Pinin expression (data not shown), indicating that other molecules or signaling pathways may contribute to Pinin's effects. Indeed, the phosphorylation status of ERK1/2 that are key molecules in MAPK pathway and well known for the growth promotion and anti-apoptotic effects in HCC, was associated with Pinin expression in GD treated HCC cells [27]. It was clear that ERK activation gradually disappeared with GD treatment in 24 h. However, in Pinin depleted cells this effect was further accelerated as it was shown that ERK phosphorylation reduced more rapidly at 12 h after GD treatment. On the contrary, Pinin overexpression partially maintained ERK activation, such effect was totally blocked by U0126, a MEK inhibitor, indicating Pinin might function at the upstream of MEK1/2. Activation of ERK was involved in many cellular processes including acceleration of HCC cell growth or resistance to metabolic stress induced apoptosis. Thus, Pinin promotes HCC cell growth and inhibits GD induced apoptosis via ERK pathway activation.

In summary, for the first time, we report that high expression of Pinin was associated with HCC development and identified the oncogenic role of Pinin in promoting growth, inhibiting apoptosis of HCC cells in GD condition through maintaining ERK phosphorylation and suppressing PARP cleavage. we propose that Pinin might be a valuable parameter in evaluating HCC risk and a novel therapeutic target for HCC treatment.

## MATERIALS AND METHODS

### Cell culture and reagents

BEL7402, SNU449, HepG2 cells were obtained from the American Type Culture Collection (Manassas, VA). BEL7402, HepG2, and LO2 cells were grown in Dulbecco's modified Eagle's medium (DMEM), supplemented with 10% fetal bovine serum (FBS), 50 U/ml penicillin, and 50 μg/ml streptomycin. SNU449 cells were grown in PRMI 1640 medium, supplemented with 10% fetal bovine serum (FBS), 50 U/ml penicillin, and 50 μg/ml streptomycin. The cells were maintained at 37°C under 5% CO_2_ in humidified air. The following antibodies were used in this study: anti-Pinin (protein tech, 18266-1-AP), anti-GAPDH (Santa Cruz Biotechnology, SC-32233), anti-ERK (Cell Signaling, 4372S), anti-phospho-ERK1/2 (Cell Signaling, 4370S), anti-PARP (Santa Cruz Biotechnology, SC-8007), and anti-Flag M2 (Sigma, F1804). U0126 (Cell Signaling, 9903) was selected as the MAPK/ERK inhibitor.

### Western blot analysis

Total protein extracted from HCC tissues and HCC cells were used for immunoblotting. In brief, cell lysates were clarified by centrifugation at 9,000 g for 10 min, and then the supernatant was collected. Protein concentration was determined using BCA Protein Assay Kit (Pierce, U.S.A). Total protein (30-60 μg) was separated on an 8% or 10% SDS-PAGE mini-gel, followed by transfering to a nitrocellulose (NC) membrane. After blocking with TTBS (50 mM Tris-HCl, 0.15 M NaCl, 0.1% Tween-20, pH 7.5) containing 5% fat-free dry milk overnight at 4°C, the membrane was incubated with antibodies and an enhanced chemiluminescence (ECL) detection system (Amersham) was used to visualize the expression of these target proteins. Three samples from each group were analyzed and the results were quantified using the Gel-Pro 4.0 analyzer software.

### Lentivirus transfection

The lentiviral transduction particles for shRNA-mediated knockdown of Pinin were purchased from Sigma (Shanghai, China). The shRNA sequences targeting Pinin were 5′-GCAGCAGAAGATTTCTTGATA-3′ (BEL7402) and 5′-GCATCGAATTTGCAGAACAAA-3′ (SNU449). The shRNA was cloned using the PLKO.1 vector. Stable knockdown cells were established as previously described [28]. The primers 5′-ATGGCGGTCGCCGTGAGAAC-3′ and 5′-TTAACGCCTTTTGTCTTTCCTGTC-3′ were used to generate plasmids encoding full-length Pinin. Pinin cDNA was then amplified by RT-PCR using total RNA from BEL7402 cells. To generate lentivirus expressing Pinin, HEK 293T cells grown on a 6 cm dish were transfected with 2 ug pCDH-Flag-Pinin or control vector, 1.5 ug psPax2, and 0.5 ug pMD2G. 24 h after the transfection, cells were cultured with DMEM containing 10% FBS for an additional 24 h. The culture medium containing lentiviral particles was centrifuged at 1,000 g for 5 min. Viral particles collected in the supernatant were used for infection. In order to establish the stable cell line, the puromycin was used as a selection marker for the infected cells. The expression efficiency was evaluated by western blot analysis.

### Cell viability assay and colony formation assay

Cell viability was detected by CCK8 assay. Cells were plated in 96-well plates at a density of 5,000 cells in 100 μl medium per well 24 h before the experiment. The cells were cultured in DMEM without glucose for 24 h, cell viability was examined by CCK8 assay. For colony formation assay, BEL7402 and SNU449 cells were trypsinized and 1,000 or 750 viable cells were subcultured in 6-well plates (in triplicate). Cells were allowed to adhere and colonize for 14 days. To visualize colonies, media was removed and cells were fixed in 96% ethanol for 10 min and stained with crystal violet staining solution.

### Detection of apoptosis

SNU449 and BEL7402 cells were seeded in 6-well culture plates (1.5×10^5^ cells/well) and incubated for 24 h at 37°C. Next, the cells were cultured in DMEM without glucose for 24 h, followed by incubation with Annexin V (Ax)-FITC and PI (10 μg/mL) at room temperature for 15 min. Finally, fluorescent intensities were determined by fluorescence activated cell sorting (FACS) using a FACSCantoII (BD, Franklin Lakes, NJ, USA).

### RNA extraction and real-time PCR

Human Hepatocellular carcinoma samples were obtained from The Second Affiliated Hospital of Dalian Medical University under the strict guidance of ethical committee. After frozen tissue samples were powdered in liquid nitrogen, Trizol was added to extract RNA. RNA quality was examined by gel electrophoresis and only paired RNA with high quality was used for following analyses.

One microgram of total RNA was used to synthesize cDNA by using the PrimeScript™ RT reagent kit (Takara, DRR037A) according to the manufacturer's instructions. Real-time PCR was performed using SYBR premix EX Taq (TaKaRa) and ROX, and analyzed with Stratagene Mx3000p (Agilent Technologies). Real-time PCR primer sequences were as follows: Actin 5′-CTCCATCCTGGCCTCGCTGT-3′ and 5′-GCTGTCACCTTCACCGTTCC-3′. Pinin 5′-GGAGGTAGAGGACGTGGTAG-3′ and 5′-TTCCT GGCGTGATTCTCTTC-3′.

### Tissue microarrays and immunohistochemistry

HCC tissue microarrays were purchased from Shanghai Outdo Biotech (Shanghai, China) and it contained 95 HCC tissues and their corresponding adjacent non-malignant normal tissues. Carcinoma tissue samples and the corresponding adjacent tissue samples were obtained in the files of Taizhou Hospital of Zhejiang Province from 2001 to 2005. The characteristics of the patients and their tumors were collected though review of medical records and pathologic reports. Informed consent with approval of the ethics committee of Taizhou Hospital of Zhejiang Province was obtained. All patients had negative histories of exposure to either chemotherapy or radiotherapy before surgery, and there was no co-occurrence of other diagnosed cancers.

Tissue samples were processed according to routine procedures. In brief, the paraffin embedded hepatocellular carcinoma tissue samples and the corresponding adjacent tissue samples were cut at 4 μm and mounted on glass slides. Then, the slides were deparaffinized, hydrated, and incubated in 3% H2O2 and microwaved to blockendogenous peroxidase activity. After 20 minutes to expose antigen hidden inside the tissue due to formalin fixation at room temperature, to inhibit non-specific antigen– antibody reactions possible in immunohistochemical staining, protein blocker was used for 5 minutes and the slides were washed thoroughly with PBS buffer. Then the slides were incubated overnight with the primary antibodies against Pinin (1:200, rabbit polyclonal antibody) at 4 centigrade. Biotinylated goat anti-rabbit secondary antibody (1:200) was applied for 20 minutes at room temperature, followed by further washing with buffer to remove unbound antibody. A complex of avidin with horseradish peroxidase was then applied for 20 minutes at room temperature. For color development, the slides were stained with 3,3′diaminobenzidine, then were counterstained with hematoxylin.

The immunostaining analysis of Pinin protein expression was assessed based on these tissue microarrays. The extent of the staining was used as criteria of evaluation. For each tissue sample, protein expression was scored according to the staining color: negative staining (no yellow), low staining (light yellow), moderate or high staining (yellowish brown or brown).

### EdU assay

The EdU incorporation assay was performed with an EdU Assay Kit (Guangzhou RIBOBIO, Guangzhou, China) according to the manufacturer's instructions. Briefly, the BEL7402 and SNU449 cells were incubated with DMEM containing 50 mM EdU for 2 h. The nuclei were also stained with Hoechst 33342 (Sigma, St Louis, MO, USA), and the images were acquired with an Olympus DP71X microscope (Olympus, Tokyo, Japan).

### Statistical analysis

Data were expressed as the mean ± SD and analyzed using unpaired 2-sided student *t* test. Statistical analysis was performed using SPSS 18.0. *P* values <0.05 were considered statistically significant and indicated as follows: *P<0.05, **P<0.01, ***P<0.001.

## SUPPLEMENTARY FIGURES


