# Supplementary material for: Pinin associates with prognosis of hepatocellular carcinoma through promoting cell proliferation and suppressing glucose deprivation-induced apoptosis
Source: Oncotarget. 2016 May 9;7(26):39694–704. doi: 10.18632/oncotarget.9233 (PMC5129963; doi:10.18632/oncotarget.9233)
Supplement: Supplementary file 1 [file oncotarget-07-39694-s001.pdf]

SUPPLEMENTARY FIGURES

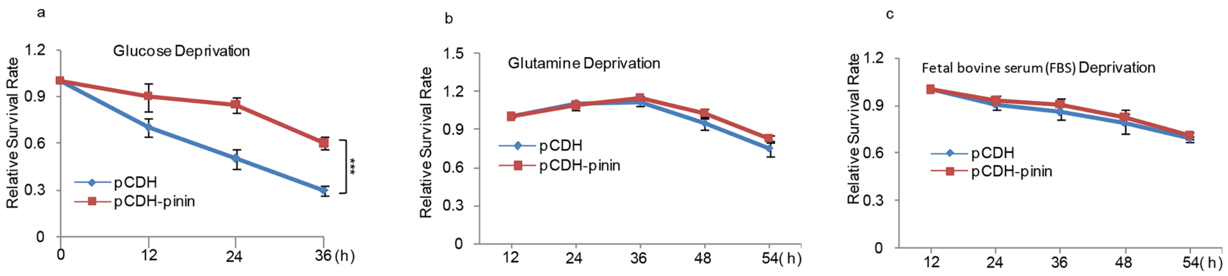

Supplementary Figure S1: a-c. Relative cell survival rates of SNU449 cells stably expressing empty vector or Pinin in the absence of glucose, glutamine, or Fetal bovine serum were measured at indicated time points. Pinin expression promoted HCC cells survival under glucose depletion and had no significant effect on cell survival under glutamine or FBS deprivation (n=3, mean ± SD, *t*-test, $^{**}P<0.01$, $^{***}P<0.001$ vs. pCDH-flag).

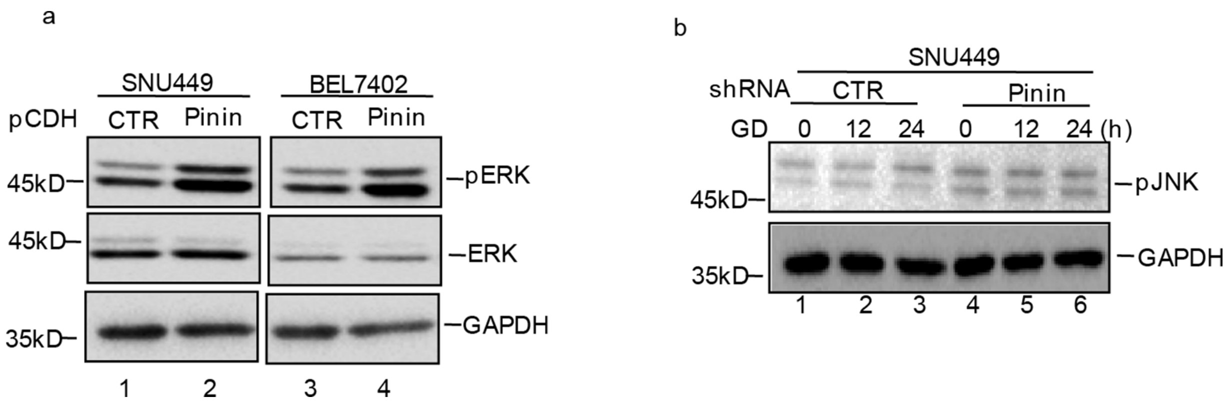

Supplementary Figure S2: a. Pinin was overexpressed in SNU449 and BEL7402 cells. Cell lysates were analyzed with the antibodies indicated. GAPDH was detected as the loading control. **b.** SNU449 cells with or without knockdown of Pinin were cultured in present of glucose or not for the indicated periods. Cell lysates were analyzed with the indicated antibodies. GAPDH was detected as the loading control.
